# (*E*)-3,4-Dihydroxy­benzaldehyde 4-ethyl­thio­semicarbazone

**DOI:** 10.1107/S1600536808009148

**Published:** 2008-04-10

**Authors:** Safa’a Fares Kayed, Yang Farina, Ibrahim Baba, Jim Simpson

**Affiliations:** a School of Chemical Sciences and Food Technology, Faculty of Science and Technology, Universiti Kebangsaan Malaysia, 43600 UKM Bangi, Selangor, Malaysia; b Department of Chemistry, University of Otago, PO Box 56, Dunedin, New Zealand

## Abstract

The title compound, C_10_H_13_N_3_O_2_S, was prepared by condensation of 3,4-dihydroxy­benzaldehyde with 4-ethyl-3-thio­semicarbazide. The mol­ecule adopts an *E* configuration with respect to the C=N bond. One of the OH substituents on the dihydroxy­benzene ring is disordered over the two possible 3-positions on either side of the ordered 4-hydr­oxy group. The occupancy of the major disorder component refined to 0.633 (7). The mol­ecule is essentially planar, with an r.m.s. deviation through all non-H atoms of 0.0862 Å. An intra­molecular N—H⋯N hydrogen bond forms between the outer amine residue and the imine N atom, generating an *S*(5) ring motif and contributing to the planarity of the mol­ecule. In the crystal structure, an extensive network of classical O—H⋯O, O—H⋯S and N—H⋯S hydrogen bonds and weak C—H⋯O and S⋯O [3.301 (3) Å] inter­actions link mol­ecules into sheets running approximately parallel to the *ab* plane.

## Related literature

For related structures, see: Swesi *et al.* (2006 ▶); Kovala-Demertzi *et al.* (2004 ▶); Jian & Li (2006 ▶). For reference structural data, see: Allen *et al.* (1987 ▶). For ring motifs, see: Bernstein *et al.* (1995 ▶).
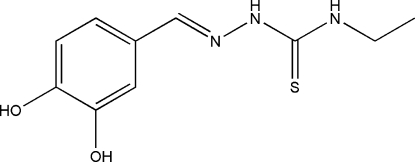


## Experimental

### 

#### Crystal data

C_10_H_13_N_3_O_2_S
*M* *_r_* = 239.29
Monoclinic, 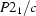

*a* = 10.6549 (12) Å
*b* = 12.9020 (16) Å
*c* = 8.6375 (11) Å
β = 107.910 (4)°
*V* = 1129.9 (2) Å^3^
*Z* = 4
Mo *K*α radiation
μ = 0.28 mm^−1^
*T* = 91 (2) K
0.44 × 0.11 × 0.09 mm

#### Data collection

Bruker APEXII CCD area-detector diffractometer
Absorption correction: multi-scan (*SADABS*; Bruker, 2006 ▶) *T*_min_ = 0.818, *T*_max_ = 0.975
12327 measured reflections
1998 independent reflections
1507 reflections with *I* > 2σ(*I*)
*R*_int_ = 0.040

#### Refinement

*R*[*F* ^2^ > 2σ(*F* ^2^)] = 0.059
*wR*(*F* ^2^) = 0.168
*S* = 1.05
1998 reflections
165 parameters
2 restraints
H atoms treated by a mixture of independent and constrained refinement
Δρ_max_ = 1.41 e Å^−3^
Δρ_min_ = −0.64 e Å^−3^

### 

Data collection: *APEX2* (Bruker 2006 ▶); cell refinement: *APEX2* and *SAINT* (Bruker 2006 ▶); data reduction: *SAINT*; program(s) used to solve structure: *SHELXS97* (Sheldrick, 2008 ▶) and *TITAN2000* (Hunter & Simpson, 1999 ▶); program(s) used to refine structure: *SHELXL97* (Sheldrick, 2008 ▶) and *TITAN2000*; molecular graphics: *SHELXTL* (Sheldrick, 2008 ▶) and *Mercury* (Macrae *et al.*, 2006 ▶); software used to prepare material for publication: *SHELXL97*, *enCIFer* (Allen *et al.*, 2004 ▶) and *PLATON* (Spek, 2003 ▶).

## Supplementary Material

Crystal structure: contains datablocks global, I. DOI: 10.1107/S1600536808009148/hg2389sup1.cif

Structure factors: contains datablocks I. DOI: 10.1107/S1600536808009148/hg2389Isup2.hkl

Additional supplementary materials:  crystallographic information; 3D view; checkCIF report

## Figures and Tables

**Table 1:** Hydrogen-bond geometry (Å, °)

*D*—H⋯*A*	*D*—H	H⋯*A*	*D*⋯*A*	*D*—H⋯*A*
N3—H3*B*⋯N1	0.88	2.23	2.626 (4)	107
O5—H5*A*⋯S1^i^	0.84	2.82	3.106 (9)	102
C2—H2⋯O5^ii^	0.95	2.65	3.335 (8)	129
N2—H2*A*⋯S1^iii^	0.88	2.52	3.392 (4)	172
O4—H4⋯O4^iv^	0.84	2.16	2.988 (5)	169
C9—H9*A*⋯O3^v^	0.99	2.46	2.985 (5)	113

Symmetry codes: (i) 
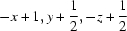
; (ii) 
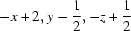
; (iii) 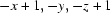; (iv) 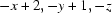; (v) 
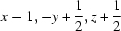
.

**Fig. 1.**
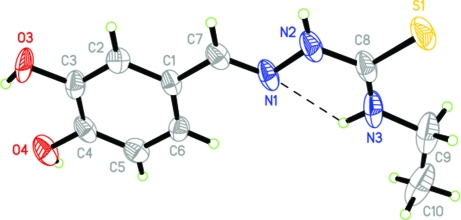
The structure of (I), with atom labels and 50% probability displacement ellipsoids for non-H atoms. The intramolecular N—H···N hydrogen bond is drawn as a dashed line. For clarity only the major disorder component of the disordered OH groups is shown.

**Fig. 2.**
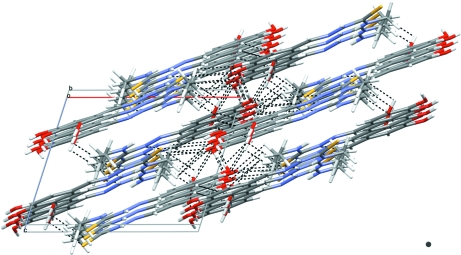
Crystal packing of (I) viewed down the b axis with hydrogen bonds drawn as dashed lines.

## supplementary crystallographic information

### Comment

For example the structure of the related molecule 2,3-dihydroxybenzaldehyde thiosemicarbazone hemihydrate has been reported by Swesi *et al.* (2006) as have the structures of a phenylthiocarbazole with a  single hydroxy-substituent on the benzylidene ring (Jian & Li, 2006) and of a palladium(II) complex of an ethylthiosemicarbonate ligand deprotonated at the phenolate ring (Kovala-Demertzi *et al.*, 2004).

The molecule adopts an *E* configuration with respect to the C=N bond and bond distances and angles are normal (Allen *et al.*, 1987). One of the OH substituents on the dihydroxy benzene ring is disordered over the two possible 3-positions (labelled O3 and O5) on either side of the ordered O4 hydroxo group. Occupancy of the O3 and H5 atoms of the major disorder component refines to 0.633 (7). The molecule is essentially planar with an r.m.s. deviation through all non-hydrogen atoms of 0.0862 Å. An intramolecular N3—H3B···N1 hydrogen bond forms between the outer amine residue and the imine N atom generating an S(5) ring motif (Bernstein *et al.*, 1995) which contributes to the planarity of the molecule.

In the crystal structure N2—H2A···S1 hydrogen bonds, Table 1, generate centrosymmetric *R*^2^_2_(8) rings. Other classical O—H···O and O—H···S hydrogen bonds combine with weak C—H···O and S1···O4^i^ interactions (*d*(S1···O4) = 3.301 (3) Å; i = -1 + *x*, 1/2 - *y*, 1/2 + *z*) to form sheets running approximately parallel to the *ac* diagonal, Fig 2.

### Experimental

The title compound C_10_H_13_N_3_O_2_S was prepared by heating an ethanolic (35 ml) solution of 3,4-dihydroxybenzaldehyde (1.4 g, 10 mmol) and 4-ethyl-3-thiosemicarbazide (1.2 g, 10 mmol) under reflux for 1 h. The resulting product was isolated and recrystallized from ethanol to afford red block-shaped crystals in 71% yield (m.p. 464–467 K).

### Refinement

The aromatic H atoms of the two disorder components were located in a difference Fourier map and refined with fixed isotropic displacement parameters with C—H distances restrained to 0.95 (1) Å. All other H-atoms were positioned geometrically and refined using a riding model with d(C—H) = 0.95 Å, *U*_iso_=1.2*U*_eq_ (C) for aromatic 0.99 Å, *U*_iso_ = 1.2*U*_eq_ (C) for CH_2_, 0.98 Å, *U*_iso_ = 1.5*U*_eq_ (C) for CH_3_ 0.88 Å, *U*_iso_ = 1.2*U*_eq_ (N) for NH and 0.84 Å, *U*_iso_ = 1.5*U*_eq_ (O) for the OH atoms. Close contacts involving the H atoms of the OH substituents, suggest that there may be unresolved disorder particularly with the location of the H atoms. The highest residual electron density peak is located at 2.56 Å from O5 and the deepest hole is located at 0.81 Å from S1.

### Crystal data

**Table:**

C_10_H_13_N_3_O_2_S	*F*_000_ = 504
*M**_r_* = 239.29	*D*_x_ = 1.407 Mg m^−^^3^
Monoclinic, *P*2_1_/*c*	Mo *K*α radiation λ = 0.71073 Å
Hall symbol: -P 2ybc	Cell parameters from 3071 reflections
*a* = 10.6549 (12) Å	θ = 2.6–25.0º
*b* = 12.9020 (16) Å	µ = 0.28 mm^−^^1^
*c* = 8.6375 (11) Å	*T* = 91 (2) K
β = 107.910 (4)º	Block, red
*V* = 1129.9 (2) Å^3^	0.44 × 0.11 × 0.09 mm
*Z* = 4	

### Data collection

**Table:**

Bruker APEXII CCD area-detector diffractometer	1998 independent reflections
Radiation source: fine-focus sealed tube	1507 reflections with *I* > 2σ(*I*)
Monochromator: graphite	*R*_int_ = 0.040
*T* = 92(2) K	θ_max_ = 25.1º
ω scans	θ_min_ = 3.1º
Absorption correction: multi-scan(SADABS; Bruker, 2006)	*h* = −12→12
*T*_min_ = 0.818, *T*_max_ = 0.975	*k* = −15→15
12327 measured reflections	*l* = −10→8

### Refinement

**Table:**

Refinement on *F*^2^	Secondary atom site location: difference Fourier map
Least-squares matrix: full	Hydrogen site location: inferred from neighbouring sites
*R*[*F*^2^ > 2σ(*F*^2^)] = 0.059	H atoms treated by a mixture of independent and constrained refinement
*wR*(*F*^2^) = 0.168	*w* = 1/[σ^2^(*F*_o_^2^) + (0.0718*P*)^2^ + 1.7231*P*] where *P* = (*F*_o_^2^ + 2*F*_c_^2^)/3
*S* = 1.05	(Δ/σ)_max_ < 0.001
1998 reflections	Δρ_max_ = 1.41 e Å^−^^3^
165 parameters	Δρ_min_ = −0.64 e Å^−^^3^
2 restraints	Extinction correction: none
Primary atom site location: structure-invariant direct methods	

### Special details

**Table:**

Geometry. All e.s.d.'s (except the e.s.d. in the dihedral angle between two l.s. planes) are estimated using the full covariance matrix. The cell e.s.d.'s are taken into account individually in the estimation of e.s.d.'s in distances, angles and torsion angles; correlations between e.s.d.'s in cell parameters are only used when they are defined by crystal symmetry. An approximate (isotropic) treatment of cell e.s.d.'s is used for estimating e.s.d.'s involving l.s. planes.
Refinement. Refinement of *F*^2^ against ALL reflections. The weighted *R*-factor *wR* and goodness of fit *S* are based on *F*^2^, conventional *R*-factors *R* are based on *F*, with *F* set to zero for negative *F*^2^. The threshold expression of *F*^2^ > σ(*F*^2^) is used only for calculating *R*-factors(gt) *etc*. and is not relevant to the choice of reflections for refinement. *R*-factors based on *F*^2^ are statistically about twice as large as those based on *F*, and *R*- factors based on ALL data will be even larger.

### Fractional atomic coordinates and isotropic or equivalent isotropic displacement parameters (Å^2^)

**Table:**

	*x*	*y*	*z*	*U*_iso_*/*U*_eq_	Occ. (<1)
C1	0.7990 (3)	0.2409 (2)	0.2636 (3)	0.0304 (7)	
C2	0.9009 (3)	0.1922 (2)	0.2235 (3)	0.0342 (7)	
H2	0.9097	0.1190	0.2323	0.041*	
C3	0.9897 (3)	0.2493 (3)	0.1712 (4)	0.0382 (8)	
H3	1.048 (11)	0.201 (8)	0.147 (17)	0.046*	0.367 (7)
O3	1.0884 (4)	0.2059 (4)	0.1330 (6)	0.0525 (14)	0.633 (7)
H3A	1.1301	0.2516	0.0999	0.079*	0.633 (7)
C5	0.8761 (3)	0.4051 (3)	0.1995 (4)	0.0394 (8)	
H5	0.862 (11)	0.477 (2)	0.177 (13)	0.047*	0.633 (7)
O5	0.8733 (10)	0.5042 (5)	0.1953 (12)	0.045 (2)	0.367 (7)
H5A	0.8856	0.5248	0.1089	0.068*	0.367 (7)
C4	0.9776 (3)	0.3557 (3)	0.1580 (4)	0.0381 (8)	
O4	1.0659 (2)	0.4115 (2)	0.1053 (3)	0.0503 (7)	
H4	1.0294	0.4656	0.0584	0.075*	
C6	0.7871 (3)	0.3484 (2)	0.2514 (3)	0.0334 (7)	
H6	0.7178	0.3825	0.2787	0.040*	
C7	0.7094 (3)	0.1775 (3)	0.3225 (3)	0.0357 (7)	
H7	0.7228	0.1047	0.3322	0.043*	
N1	0.6134 (2)	0.2175 (2)	0.3612 (3)	0.0390 (7)	
N2	0.5403 (3)	0.1489 (3)	0.4216 (3)	0.0442 (7)	
H2A	0.5567	0.0819	0.4244	0.053*	
C8	0.4433 (3)	0.1862 (3)	0.4763 (4)	0.0458 (9)	
S1	0.36442 (11)	0.10262 (10)	0.56744 (13)	0.0685 (4)	
N3	0.4168 (3)	0.2853 (3)	0.4565 (3)	0.0506 (8)	
H3B	0.4660	0.3233	0.4133	0.061*	
C9	0.3112 (4)	0.3371 (4)	0.5010 (5)	0.0678 (13)	
H9A	0.3118	0.3133	0.6102	0.081*	
H9B	0.2252	0.3177	0.4224	0.081*	
C10	0.3260 (5)	0.4506 (5)	0.5024 (6)	0.0804 (15)	
H10A	0.4042	0.4708	0.5917	0.121*	
H10B	0.2476	0.4831	0.5179	0.121*	
H10C	0.3361	0.4735	0.3987	0.121*	

### Atomic displacement parameters (Å^2^)

**Table:**

	*U*^11^	*U*^22^	*U*^33^	*U*^12^	*U*^13^	*U*^23^
C1	0.0251 (14)	0.0466 (18)	0.0195 (14)	−0.0048 (12)	0.0069 (11)	0.0028 (12)
C2	0.0353 (16)	0.0376 (17)	0.0304 (15)	0.0009 (13)	0.0112 (13)	0.0039 (13)
C3	0.0292 (16)	0.057 (2)	0.0319 (16)	0.0021 (14)	0.0146 (13)	0.0001 (15)
O3	0.037 (3)	0.075 (3)	0.059 (3)	0.009 (2)	0.036 (2)	−0.002 (2)
C5	0.0427 (18)	0.0392 (18)	0.0365 (17)	−0.0057 (15)	0.0122 (14)	0.0051 (15)
O5	0.048 (4)	0.035 (4)	0.053 (5)	−0.010 (4)	0.015 (3)	0.013 (4)
C4	0.0331 (16)	0.056 (2)	0.0265 (15)	−0.0149 (15)	0.0113 (13)	0.0037 (14)
O4	0.0449 (14)	0.0657 (17)	0.0471 (14)	−0.0192 (12)	0.0241 (12)	0.0093 (12)
C6	0.0304 (15)	0.0422 (17)	0.0295 (16)	0.0020 (13)	0.0119 (13)	0.0009 (13)
C7	0.0348 (16)	0.0476 (19)	0.0252 (15)	−0.0110 (14)	0.0099 (13)	0.0018 (13)
N1	0.0281 (13)	0.0639 (18)	0.0267 (13)	−0.0131 (12)	0.0110 (11)	0.0059 (12)
N2	0.0380 (14)	0.0675 (19)	0.0319 (14)	−0.0205 (14)	0.0178 (12)	−0.0021 (13)
C8	0.0331 (17)	0.082 (3)	0.0250 (16)	−0.0237 (18)	0.0133 (13)	−0.0102 (17)
S1	0.0738 (7)	0.0931 (9)	0.0585 (7)	−0.0513 (6)	0.0497 (6)	−0.0299 (6)
N3	0.0305 (14)	0.092 (3)	0.0352 (15)	−0.0037 (15)	0.0181 (12)	0.0076 (16)
C9	0.038 (2)	0.131 (4)	0.039 (2)	0.008 (2)	0.0176 (16)	0.000 (2)
C10	0.076 (3)	0.122 (5)	0.053 (3)	0.036 (3)	0.034 (2)	0.000 (3)

### Geometric parameters (Å, °)

**Table:**

C1—C2	1.388 (4)	C7—N1	1.278 (4)
C1—C6	1.395 (5)	C7—H7	0.9500
C1—C7	1.462 (4)	N1—N2	1.384 (3)
C2—C3	1.380 (4)	N2—C8	1.350 (4)
C2—H2	0.9500	N2—H2A	0.8800
C3—O3	1.319 (5)	C8—N3	1.310 (5)
C3—C4	1.380 (5)	C8—S1	1.702 (3)
C3—H3	0.950 (10)	S1—O4^i^	3.301 (2)
O3—H3A	0.8400	N3—C9	1.457 (5)
C5—O5	1.279 (7)	N3—H3B	0.8800
C5—C6	1.376 (4)	C9—C10	1.473 (7)
C5—C4	1.394 (5)	C9—H9A	0.9900
C5—H5	0.950 (10)	C9—H9B	0.9900
O5—H5A	0.8400	C10—H10A	0.9800
C4—O4	1.369 (3)	C10—H10B	0.9800
O4—H4	0.8400	C10—H10C	0.9800
C6—H6	0.9500		
			
C2—C1—C6	119.4 (3)	N1—C7—C1	121.7 (3)
C2—C1—C7	118.6 (3)	N1—C7—H7	119.1
C6—C1—C7	121.9 (3)	C1—C7—H7	119.1
C3—C2—C1	120.6 (3)	C7—N1—N2	115.4 (3)
C3—C2—H2	119.7	C8—N2—N1	119.0 (3)
C1—C2—H2	119.7	C8—N2—H2A	120.5
O3—C3—C4	117.6 (3)	N1—N2—H2A	120.5
O3—C3—C2	122.3 (4)	N3—C8—N2	117.4 (3)
C4—C3—C2	120.1 (3)	N3—C8—S1	124.2 (3)
C4—C3—H3	133 (8)	N2—C8—S1	118.4 (3)
C2—C3—H3	107 (8)	C8—N3—C9	124.5 (3)
C3—O3—H3A	109.5	C8—N3—H3B	117.8
O5—C5—C6	121.9 (5)	C9—N3—H3B	117.8
O5—C5—C4	117.6 (5)	N3—C9—C10	111.6 (4)
C6—C5—C4	120.5 (3)	N3—C9—H9A	109.3
C6—C5—H5	120 (7)	C10—C9—H9A	109.3
C4—C5—H5	119 (7)	N3—C9—H9B	109.3
C5—O5—H5A	109.5	C10—C9—H9B	109.3
O4—C4—C3	119.6 (3)	H9A—C9—H9B	108.0
O4—C4—C5	120.8 (3)	C9—C10—H10A	109.5
C3—C4—C5	119.6 (3)	C9—C10—H10B	109.5
C4—O4—H4	109.5	H10A—C10—H10B	109.5
C5—C6—C1	119.9 (3)	C9—C10—H10C	109.5
C5—C6—H6	120.1	H10A—C10—H10C	109.5
C1—C6—H6	120.1	H10B—C10—H10C	109.5

Symmetry codes: (i) *x*−1, −*y*+1/2, *z*+1/2.

### Hydrogen-bond geometry (Å, °)

**Table:**

*D*—H···*A*	*D*—H	H···*A*	*D*···*A*	*D*—H···*A*
N3—H3B···N1	0.88	2.23	2.626 (4)	107
O5—H5A···S1^ii^	0.84	2.82	3.106 (9)	102
C2—H2···O5^iii^	0.95	2.65	3.335 (8)	129
N2—H2A···S1^iv^	0.88	2.52	3.392 (4)	172
O4—H4···O4^v^	0.84	2.16	2.988 (5)	169
C9—H9A···O3^i^	0.99	2.46	2.985 (5)	113

Symmetry codes: (ii) −*x*+1, *y*+1/2, −*z*+1/2; (iii) −*x*+2, *y*−1/2, −*z*+1/2; (iv) −*x*+1, −*y*, −*z*+1; (v) −*x*+2, −*y*+1, −*z*; (i) *x*−1, −*y*+1/2, *z*+1/2.
